# Photo Quiz: An uncommon gram-negative pathogen in a splenectomized child with sickle cell disease

**DOI:** 10.1128/jcm.01209-25

**Published:** 2026-04-30

**Authors:** Jessy Zenon, Hosam Dawoud, Yasmine Benhadid-Brahmi, Florence Missud, Marie Parizot, André Birgy, Stéphane Bonacorsi, Audrey Baron

**Affiliations:** 1 Service de Microbiologie, Hôpital Robert Debré, AP-HP, , Paris, France; 2 Université Paris Cité, IAME, INSERM 555089, Paris, France; 3 Referral Center for Sickle Cell Disease, Hematology Unit, Robert Debre Hospital, AP-HP 26930 , , Paris, France; 4 Equipe Opérationnelle d'Infectiologie, Service de Pédiatrie Générale, Hôpital Robert Debré, AP-HP, , Paris, France; Mayo Clinic Minnesota, Rochester, Minnesota, USA

## PHOTO QUIZ 

An 8-year-old boy with homozygous sickle cell disease (HbSS) and a history of splenectomy presented to the emergency department with fever, pallor, and a headache unresponsive to paracetamol and ibuprofen over the past 24 h. He was receiving hydroxycarbamide and was born in France to Ivorian parents, with no history of travel abroad.

On examination, his temperature was 40°C, and pulmonary, cardiac, and neurological assessments were unremarkable. There were no urinary symptoms, meningeal signs, skin rashes, purpura, or pain suggestive of a vaso-occlusive crisis.

Initial laboratory tests revealed leukocytosis at 21 ×10^9^/L (reference range: 4.5–13.5 × 10^9^/L), including a neutrophil count of 17.2 × 10^9^/L (2–8 × 10^9^/L), a platelet count of 326 × 10^9^/L (150–450 × 10^9^/L), and a hemoglobin concentration of 6.2 g/dL (11.5–15.5 g/dL). Inflammatory markers were elevated: C-reactive protein of 84 mg/L (<10 mg/L) and procalcitonin of 6 µg/L (<0.5 µg/L). Liver function tests showed alanine aminotransferase of 27 U/L (5–45 U/L), aspartate aminotransferase of 84 U/L (5–45 U/L), total bilirubin of 112 µmol/L (<17 µmol/L), and direct bilirubin of 14 µmol/L (<5 µmol/L).

At admission, only one BD BACTEC Plus Aerobic blood culture bottle was collected (no anaerobic bottle was obtained due to the difficulty of venous access). The patient subsequently received a transfusion of 325 mL of packed red blood cells and was started on intravenous cefotaxime at 50 mg/kg (body weight, 48 kg), corresponding to a dose of 2.4 g every 6 h.

**Fig 1:**
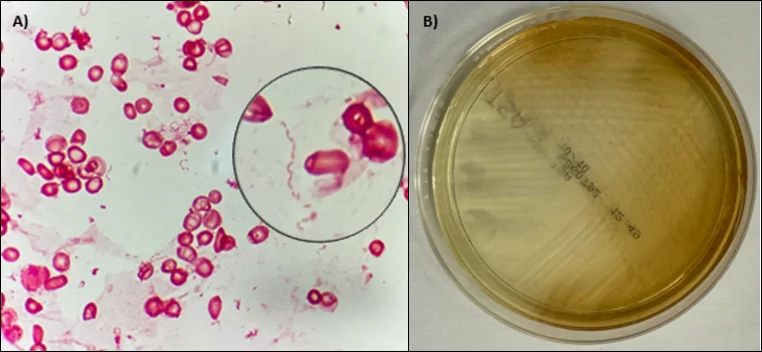
(**A**) Gram stain of the positive blood culture showing gram-negative bacilli. (**B**) Brown-pigmented colonies on Tryptone Soya Agar after 48 h of incubation.

He remained hospitalized for 2 days for monitoring and was then discharged because of clinical improvement. However, the blood culture flagged positive for gram-negative coccobacilli after 46 h of incubation, shortly after the patient’s discharge, while the BCID FilmArray panel (BioFire, USA) remained negative. The microbiologist informed the emergency physician of the result by phone, and the patient was subsequently readmitted. The organism grew on chocolate agar and non-selective Tryptone Soya Agar (Thermo Fisher Scientific, USA) within 48 h (Fig. 1).

What is your diagnosis?

## ANSWER TO THE PHOTO QUIZ

### Diagnosis: bacteriemia caused by *Bordetella holmesii*

*B. holmesii* is a gram-negative bacillus closely related to *B. pertussis*, first described in 1983 (1). Microscopically, it typically appears as chain-forming coccobacilli (Fig. 1A) and grows slowly on both routine and enriched media. *B. holmesii* is oxidase-negative, non-saccharolytic, urease-negative, and non-hemolytic on blood agar. A distinctive feature is its brown pigmentation, visible after approximately 48 h of incubation (at 35 +/– 2°C) on Tryptone Soya Agar or other non-enriched media (2) (Fig. 1B). Species identification was confirmed using MALDI-TOF mass spectrometry (Bruker Biotyper, Germany), with score values ranging from 1.82 to 1.92 (score category B+), consistent with prior reports indicating that *B. holmesii* often yields lower identification scores than *B. pertussis*, yet remains correctly identified (3).

Clinically, *B. holmesii* can cause a pertussis-like respiratory illness but lacks the pertussis toxin gene, which could explain shorter clinical course. PCR assays targeting IS481, which is present in *B. holmesii* genome, may therefore misidentify *B. holmesii* as *B. pertussis* (4). Invasive infections caused by B. holmesii, including bacteremia, endocarditis, and meningitis, are rare and occur almost exclusively in patients who are anatomically or functionally asplenic, or otherwise immunocompromised. Most bacteremia cases have been reported in asplenic individuals (5, 6).

Standardized antimicrobial susceptibility testing methods and interpretive breakpoints are not established, and published susceptibility profiles remain heterogeneous. A literature review by Pittet et al. suggests that broad-spectrum beta-lactams, including third-generation cephalosporins (cefotaxime and ceftriaxone), may be ineffective against *B. holmesii*, whereas lower MICs are generally observed with carbapenems, fluoroquinolones, cefepime, ceftazidime, and macrolides (1).

In our case, MICs were determined by E-test (Liofilchem, Italy) on Mueller–Hinton F agar (Bio-Rad, France). The results were as follows (µg/mL): amoxicillin, 1.0; cefotaxime, 32; ceftazidime, 0.5; ciprofloxacin, 0.125; azithromycin, 0.25; and rifampicin, 0.5, consistent with prior reports (7). The patient initially received cefotaxime, then was switched to ceftazidime for 3 days, followed by oral amoxicillin (2 g three times daily for seven days), with full clinical recovery.

### Key point

Although *B. holmesii* is rarely encountered in clinical practice, its isolation from blood cultures, particularly in asplenic patients, should be considered clinically significant. Given its intrinsic resistance to cefotaxime/ceftriaxone, empirical antibiotic choices for febrile episodes in asplenic or immunocompromised individuals should be chosen with care.
